# Altered Spontaneous Brain Activity and Functional Integration in Hemodialysis Patients With End-Stage Renal Disease

**DOI:** 10.3389/fneur.2021.801336

**Published:** 2022-02-09

**Authors:** Huanhuan Su, Shishun Fu, Mengchen Liu, Yi Yin, Kelei Hua, Shandong Meng, Guihua Jiang, Xianyue Quan

**Affiliations:** ^1^Department of Radiology, Zhujiang Hospital, Southern Medical University, Guangzhou, China; ^2^Department of Medical Imaging, Guangdong Second Provincial General Hospital, Guangzhou, China; ^3^Department of Organ Transplantation, Guangdong Second Provincial General Hospital, Guangzhou, China

**Keywords:** end-stage renal disease, functional connectivity, amplitude of low-frequency fluctuations, hemodialysis, resting-state fMRI

## Abstract

**Purpose:**

Using the amplitude of low-frequency fluctuation (ALFF) and functional connectivity (FC) algorithm to study the alteration of brain function in hemodialysis patients with end-stage renal disease (ESRD).

**Patients and Methods:**

We recruited 20 patients with ESRD on regular hemodialysis and 17 healthy controls (HCs). All of the participants underwent resting-state fMRI (rs-fMRI), neuropsychological tests, and blood biochemical examination. The individual ALFF values between the two groups were tested by an independent sample *t*-test. Then, we set the altered ALFF brain areas as seed regions of interest (ROIs), and FC analysis was used to investigate the functional integration patterns between the seed ROI and the voxels within the whole brain.

**Results:**

The ALFF values of the right precuneus and angular gyrus (RAG) in the ESRD group were lower than those in the HC subjects, but the right precentral gyrus showed higher ALFF values in patients. Hemoglobin (Hb) was negatively correlated with the ALFF values of the right precentral gyrus, and the ALFF values of the right precuneus were negatively correlated with line-tracing test (LTT) scores in patients with ESRD. Patients with ESRD show decreased connectivity between the RAG and the left precuneus, right superior frontal gyrus (RSFG), and the connectivity within the RAG was weak. In addition, FC in the RAG-right cuneus, right precuneus-left supramarginal gyrus was enhanced in the patient group.

**Conclusion:**

Our research suggested that, in hemodialysis patients with ESRD, the brain areas with abnormal spontaneous brain activity and FC are mainly located in the default mode network (DMN) regions. Hb and the LTT results were correlated with abnormal spontaneous brain activity. These findings provide additional evidence to understand the possible underlying neuropathological mechanisms in patients with ESRD.

## Introduction

Cognitive impairment is common in patients with end-stage renal disease (ESRD), especially in those who have received hemodialysis (1–3). These cognitive deficits include concentration decline (4), executive disorder, cognitive slowing, disorientation, and memory and language disturbance (5–7). This might affect the ability of patients to undergo cooperative treatment and reduce their life. However, clinicians often ignore these cognitive disorders in hemodialysis patients with ESRD. Patients with ESRD may develop cognitive dysfunction before any obvious neurological symptoms are observed. Therefore, studying the cognitive impairment pattern of hemodialysis patients with ESRD, and elucidating its pathophysiological mechanism, can help to prevent disease progression and ameliorate the prognosis of the patients.

Magnetic resonance imaging is a non-invasive technique, and plays a significant role in revealing the neuropathological mechanism of cognitive dysfunction. Recently, researchers have used many MRI methods to study the neuropathological changes in patients with ESRD, including structural, functional, and perfusion MRI (8–16). By using various resting-state fMRI (rs-fMRI) algorithms, they concentrated more on the spontaneous activity of the neuronal networks in patients with ESRD. Among these rs-fMRI algorithms, the amplitude of low-frequency fluctuation (ALFF) has been regarded as an effective tool to reflect the internal brain reactions at the baseline status, and it can measure the magnitude or amplitude of spontaneous brain activity directly (17). Therefore, we selected the altered ALFF brain areas as seeds for functional connectivity (FC) analysis. The FC algorithm supplies an invaluable tool for identifying resting-state neuronal networks between brain regions and illuminating the altered brain activity from the perspective of functional integration (18). Thus, studies combining ALFF and FC algorithms can help us to understand the relationship between complex brain function and cognitive impairment. In the recent years, this method has been applied to polycystic ovary syndrome (19), anti-N-methyl-D-aspartate receptor encephalitis (20), and temporal glioma (21).

To our knowledge, previous ALFF studies found that patients with ESRD have abnormal spontaneous brain activity (22–24). For instance, Luo et al. found that the ALFF values of the left inferior parietal lobe (IPL), left superior parietal lobe, and left precuneus in peritoneal dialysis patients with ESRD were reduced (24). Moreover, many studies using whole-brain voxel-wised and seed-wised FC analysis have found that FC in the default mode network (DMN) was impaired in patients with ESRD (25–28). Some of these studies performed combined use of voxel-based morphometry (VBM) and FC to study the alteration of intrinsic brain activity in patients with ESRD. Ding et al. indicated that the FC between putamen and supplementary motor area (SMA) was decreased, which was correlated with sensorimotor abnormalities and lower hemoglobin (Hb) in patients with ESRD (26). Jin et al. found that functional integrations in the thalamocortical network and the connection within the basal-ganglia were decreased in hemodialysis patients with ESRD (27). Chen et al. studied the regional homogeneity (ReHo) and FC alteration in patients with ESRD, and they found that hemodialysis may impair the cognitive function of patients with ESRD (25). They used different methods to indicate that patients with ESRD had abnormal functional integration. However, there are few reports on the combined use of ALFF and FC to study the abnormal brain function in patients with ESRD.

We hypothesize that hemodialysis patients with ESRD may have abnormal spontaneous brain activity and functional integration impairment at the same time. The purpose of our research was to study the functional connectivity patterns between the brain regions with altered ALFF value and the voxels within the whole brain in hemodialysis patients with ESRD, and to analyze the correlation between the results and clinical biomarkers.

## Materials and Methods

### Subjects

This prospective study was approved by the Ethics Committee of Guangdong Second Provincial General Hospital. Written informed consent was obtained from all the subjects before they were included in this research. We enrolled 20 patients with ESRD from the Department of Organ Transplantation in our hospital and 17 healthy volunteers from the community. All of the subjects were younger than 50 years in order to avoid possible confounding factors. All of the patients were diagnosed as stage 5 of chronic kidney disease, the glomerular filtration rate was almost completely reduced (<15 ml/min/1.73 m^2^). The patients were required to have received treatment with conventional hemodialysis for over three months. The etiology and hemodialysis duration were collected from electronic medical records of the patients.

We excluded patients with drug abuse, diabetes, mental illness, alcoholism, major neurological diseases (such as stroke, severe head injury, visible lesions, or epilepsy), left-handedness, head rotation >1.0°, or translation >1.0 mm during MR scanning. All of the patients completed blood biochemical tests within 24 h before MR examination, including serum creatinine, uric acid, urea levels, Hb, Kalium (K^+^), phosphate (P), and calcium (Ca^2+^). Before MRI examination, all of the subjects were asked to complete a questionnaire, including age, sex, years of education, and a series of neuropsychological tests.

The neuropsychological tests included Self-rating Depression Scale (SDS), Self-rating Anxiety Scale (SAS), digit symbol test (DST), line tracing test (LTT), number connection test type A and B (NCT-A/B), serial dotting test (SDT), and Montreal Cognitive Assessment (MoCA). The SAS and SDS were used to exclude patients with anxiety and depression. The sensitivity of the MoCA scale in screening mild cognitive impairment is higher than Mini-mental State Examination (MMSE) (29). NCT-A/B, DST, SDT, and LTT assess attention, psychomotor speed, and visual memory (30).

### Magnetic Resonance Imaging Data Acquisition

Magnetic resonance imaging data were obtained using a 3.0 Tesla MR scanner (Ingenia; Philips, Best, the Netherlands) in the Department of Medical Imaging, Guangdong Second Provincial General Hospital. All of the subjects were instructed to keep their eyes closed and awake, and to not think of anything systematically during MR scanning. In order to detect clinically silent lesions, conventional imaging sequences (such as T1-weighted images and T2-FLAIR images) were performed for every subject. The rs-fMRI data were collected using the echo-planar image (EPI) sequence, which is sensitive to blood oxygenation level-dependent (BOLD) contrast. The parameters of EPI sequence were as follows: repetition time (TR) = 2,000 ms, echo time (TE) = 30 ms, flip angle = 90°, field-of-view (FOV) = 230 × 230 mm^2^, matrix = 64 × 64, and total volume = 240. There were 33 axial slices with 3.5 mm thickness and 0.7 intersection gap in total. The duration of each rs-fMRI scan was 8 min. All of the subjects were asked several questions intended to check the extent of their cooperation after scanning.

### Data Preprocessing

Image preprocessing was conducted using a MATLAB toolbox known as Data Processing Assistant for Resting-State [DPARSF (31); http://restfmri.net/forum/DPARSF]. Because the signal is unstable because of an incomplete T1 relaxation at the beginning of acquisition, and the subject needs to adapt to the scanning state, the first 10 time points of each set of fMRI data need to be removed. There were only 230 volumes which were left for analysis. Next, the slice timing, head motion realignment, and spatial normalization to the standard Montreal Neurological Institute (MNI) template with a resampled voxel size of 3 × 3 × 3 mm^3^ were conducted. The maximum translation of head movement in any of the x, y, or z direction of all subjects was <1.0 mm, or the maximum rotation about three axes during scanning were <1.0°. In our study, one healthy control (HC) and three patients were eliminated because of an excessive head motion. And then, the normalized data were smoothed with a 4-mm full-width at half maximum. Finally, nuisance covariates were regressed out from the fMRI data, including global mean signals, head motion parameters, cerebrospinal fluid signals, and white matter signals.

### Amplitude of Low-Frequency Fluctuation Analysis

Amplitude of low-frequency fluctuation analysis was performed by using a similar toolkit as previously described (17). After preprocessing, the time series for each voxel was filtered to remove the effects of high-frequency noise (such as respiratory and heart rhythms) and very low frequency drift. The linear trend removed for the fatigue of subjects and the high temperature of the magnetic resonance scanner caused by long-time scanning. Next, the power spectrum was obtained by transforming the filtered time series to the frequency domain with a fast Fourier transformation. The square root of the power spectrum was calculated and averaged throughout 0.01–0.1 Hz at each voxel. This averaged square root was considered to be the ALFF. Divided the ALFF of each voxel by the global mean ALFF value for standardization. The standardized ALFF value of each given voxel was used for statistical analysis.

### Functional Connectivity Analysis

Seed-based FC analysis was used in our study. The regions showed that ALFF differences between patients with ESRD and controls were selected as regions of interest (ROIs) for FC analysis. The fMRI time series of all voxels within the region with abnormal ALFF were averaged to obtain the reference time series of each ROI. Next, a bandpass filter (0.01–0.1 Hz) was performed for each time series. The Pearson's correlation coefficients of the time course between each seed and each voxel of the whole brain were calculated. Fisher's r to z transformation was used to improve the normality of the correlation coefficients.

### Statistical Analysis

The statistical analysis of clinical and demographic data were carried out by the software SPSS version 22.0 (Chicago, IL, USA). Independent two-sample *t*-tests were used to analyze the differences in age, education level, MoCA, SDS, SAS, DST, NCT-A/B, LTT, and SDT between patients and HCs. The difference in sex was calculated by the chi-squared test.

Taking age, duration of education, and sex as confounding covariates, voxel-by-voxel two-sample *t*-test was used to assess the ALFF differences between patients and HCs (FDR corrected, *p* < 0.05). A partial correlation coefficient was used to analyze the association between ALFF values and neuropsychological test scores, and chemical results in the patient group. Age, sex, and duration of education were set as control variables. *P*-value < 0.05 was considered to be significantly correlated (two-tail test, Bonferroni corrected).

A one-sample *t*-test was applied to analyze the Z score FC maps within the ESRD group and the healthy group (*p* < 0.05, FDR corrected). Then, compared the Z score FC maps between patients with ESRD and HCs by using two-sample *t*-tests, with the threshold of significance differences set at *p* < 0.05 using FDR correction in the REST software (http://www.restfmri.net). To study the association between FC intensity of regions and neuropsychological test scores, and chemical results in patients with ESRD, we also used the Partial correlation analysis as aforementioned (two-tail test, Bonferroni corrected).

## Results

### Demographic Results

The results of demographic, neuropsychological, and blood biochemical tests are shown in Table 1. There was no significant difference in age, sex, education, SAS, SDS, NCT-A/B, SDT, DST, and LTT between the patient and control groups (all *P* > 0.05). The score in the MoCA of the ESRD group was lower than that of the HC group (*p* < 0.05).

**Table 1:** Demographic and test results of patients and HC.

	**ESRD (*n* = 20)**	**HC (*n* = 17)**	***P*-value**
Age (years)	37.1 ± 8.6	38.5 ± 6.9	0.573
Sex (males/females)	15/5	13/4	0.917
Education (years)	12.2 ± 2.8	13.2 ± 3.1	0.294
Hemodialysis duration (months)	13.4 ± 12.0	/	/
SAS (score)	31.8 ± 5.3	29.9 ± 4.6	0.265
SDS (score)	33.4 ± 5.3	31.2 ± 5.2	0.209
MoCA (score)	25.4 ± 1.7	26.6 ± 1.5	0.030
NCT-A (s)	46.8 ± 13.4	39.7 ± 14.0	0.125
NCT-B (s)	81.5 ± 31.9	66.8 ± 32.4	0.175
DST (score)	47.2 ± 9.7	54.4 ± 13.5	0.071
SDT (s)	44.3 ± 9.4	38.9 ± 7.5	0.064
LTT (s)	45.1 ± 12.1	43.7 ± 10.3	0.530
Creatinine (μmol/L)	1038.6 ± 250.5	/	/
Urea (μmol/L)	21.6 ± 4.2	/	/
Uric acid (μmol/L)	453.2 ± 114.3	/	/
Hb (g/L)	98.4 ± 19.8	/	/
K^+^ (mmol/L)	4.42 ± 0.6	/	/
P (mmol/L)	2.24 ± 0.64	/	/
Ca^2+^ (mg/L)	2.37 ± 0.22	/	/

*Except for sex, other measurement data are presented as mean ± SD. ESRD, end-stage renal disease; HC, healthy control; SAS, self-rating anxiety scale; SDS, self-rating depression scale; MoCA, montreal cognitive assessment; NCT-A, number connection test A; NCT-B, number connection test B; SDT, serial dotting test; DST, digital symbol test; LTT, line tracing test; Hb, hemoglobin; K^+^, kalium; P, phosphorus; Ca^2+^, calcium*.

### ALFF Analysis

**Figure 1:**
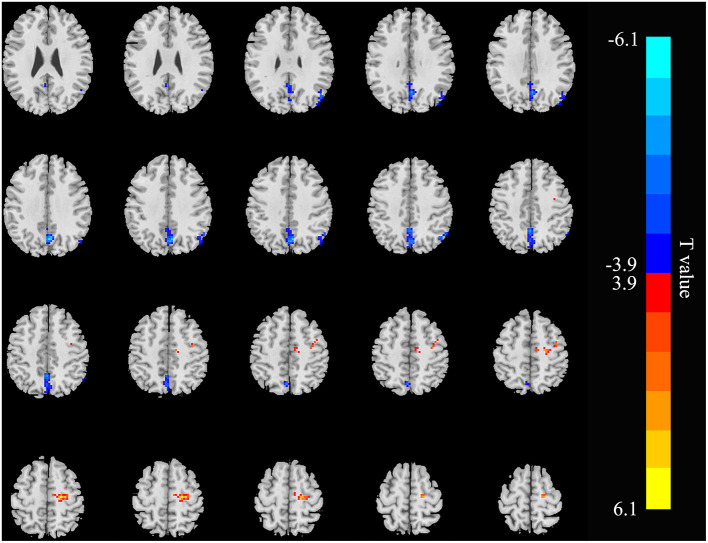
Brain areas showing ALFF differences in the patients with end-stage renal disease (ESRD) relative to controls (*p* < 0.05, FDR corrected). Patients with ESRD showed significantly decreased ALFF values in the right angular gyrus and precuneus. But the right precentral gyrus showed increased ALFF values in patients with compared with controls. ALFF, amplitude of low-frequency fluctuation; ESRD, end-stage renal disease. The color bar represents *t*-values, which is obtained by the two-sample *t-*test. The reduced ALFF value is represented by a cool color, while the increased ALFF value is expressed in warm color.

**Figure 2:**
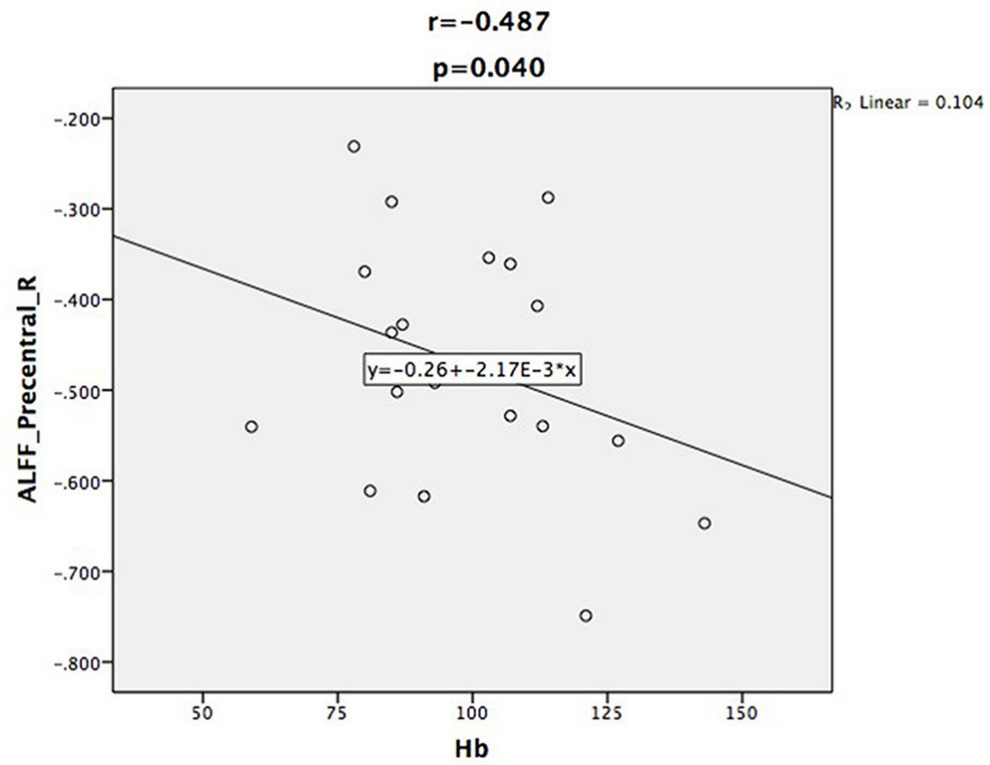
Scatter plot shows the correlation between ALFF values of the right precentral gyrus with Hb in the patient group. The ALFF values of the right precentral gyrus were negatively correlated with Hb (*r* = −0.487, *p* = 0.040).

**Figure 3:**
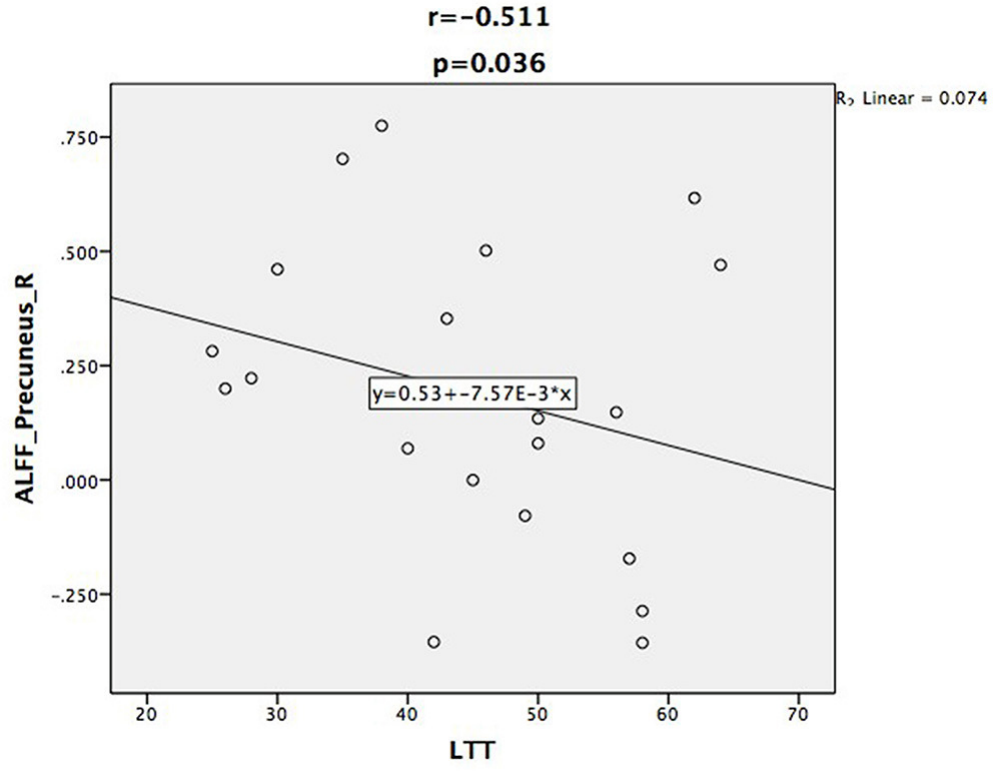
Scatter plot shows the correlation between ALFF values of the right precuneus with LTT scores. The ALFF values of the right precuneus were negatively correlated with LTT scores (*r* = −0.511, *p* = 0.036).

Relative to controls, the ALFF values of the right precuneus and angular gyrus (RAG) were significantly decreased in patients with ESRD. But the right precentral gyrus exhibited increased ALFF values in patients with ESRD compared with controls (Figure 1; Table 2). The ALFF values of the right precentral gyrus were negatively correlated with Hb (*r* = −0.487, *p* = 0.040, Figure 2), and the ALFF values of the right precuneus were negatively correlated with LTT results in patients with ESRD (*r* = −0.511, *p* = 0.036, Figure 3).

**Table 2:** Brain areas with different amplitude of low-frequency fluctuation (ALFF) values in the patients with end-stage renal disease (ESRD) relative to the controls.

**Brain areas**	**Voxel**	**Peak T score**	**MNI coordinates**		
			**x**	**y**	**z**
Right angular gyrus	51	−4.8195	45	−63	42
Right precuneus	162	−6.0136	3	−66	36
Right precentral gyrus	67	5.6244	21	−18	57

### Functional Connectivity Analysis

**Figure 4:**
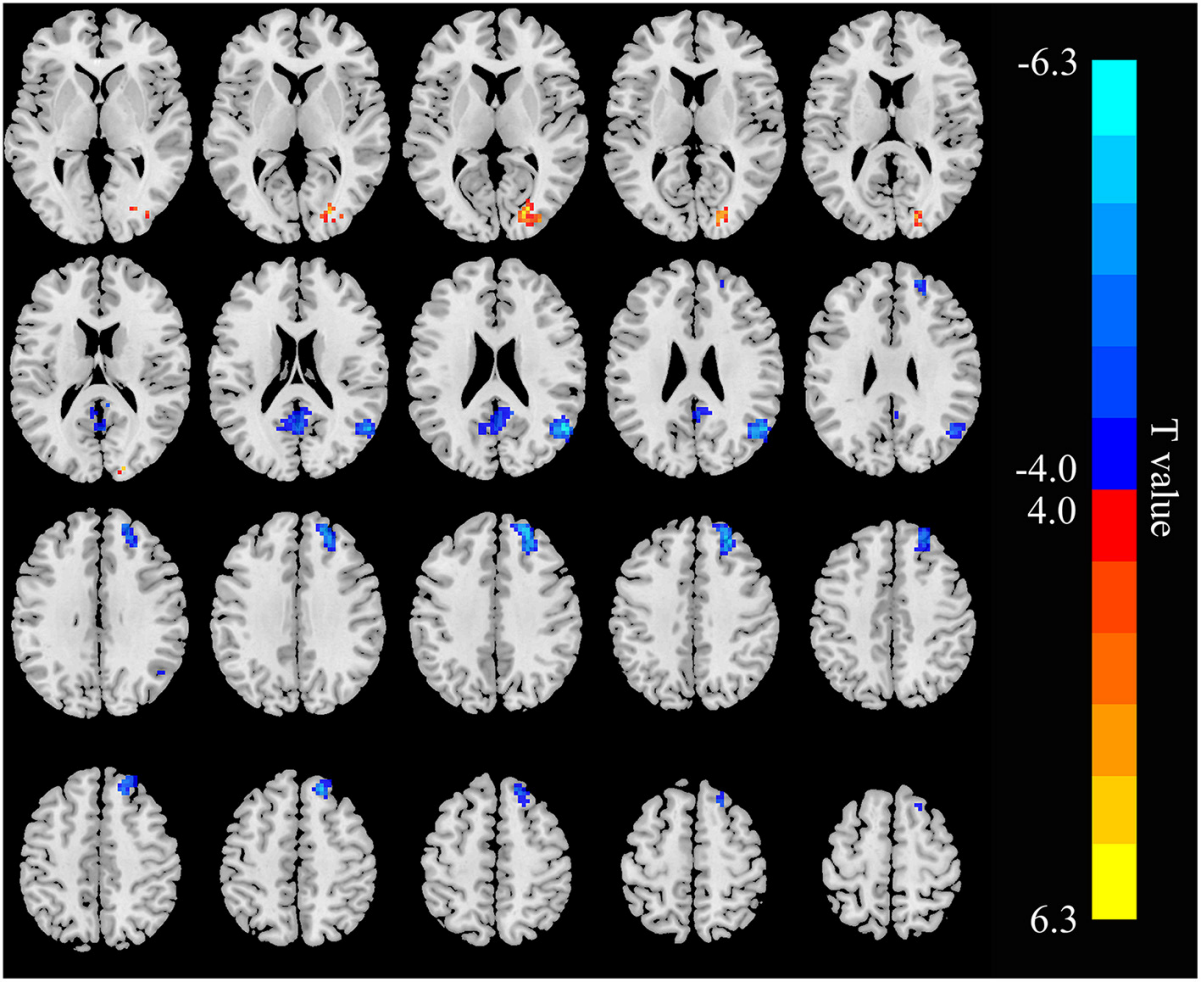
Functional connectivity (FC) differences in the right angular gyrus between patients with ESRD and HC (*p* < 0.05, FDR corrected). Patients with ESRD show reduced connectivity between the right-angular gyrus with the left precuneus and right superior frontal gyrus, and increased connectivity between the right angular gyrus and right cuneus; the connectivity within the right angular gyrus was weak.

**Figure 5:**
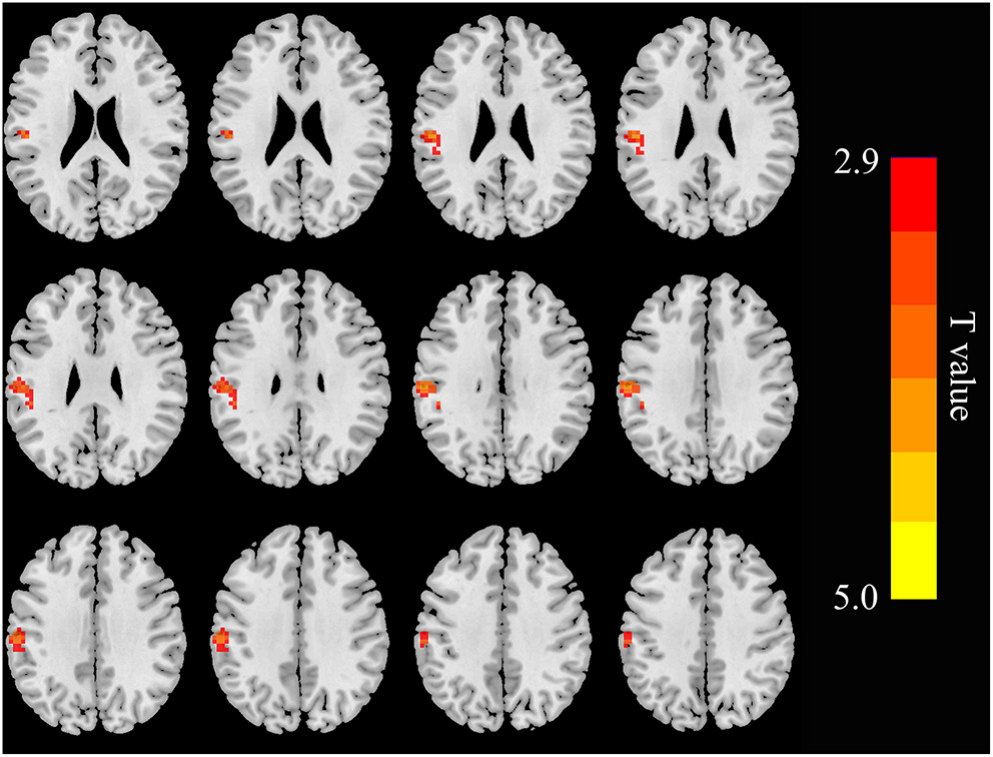
Functional connectivity differences in the right precuneus between patients with and HC (*p* < 0.05, FDR corrected). Patients with ESRD show increased connectivity between the right precuneus with the left supramarginal gyrus.

The three seed ROIs, in which increased/decreased ALFF values were found in hemodialysis patients with ESRD, were chosen for FC analysis. Relative to the control group, the patient group showed decreased connectivity between the RAG and the left precuneus, right superior frontal gyrus (RSFG), but increased connectivity between the RAG and right cuneus; the connectivity within the RAG was weak (Figure 4; Table 3). In addition, connectivity between the right precuneus and left supramarginal gyrus was enhanced in the ESRD group (Figure 5; Table 4). When setting the right precentral gyrus as seed ROI, patients with ESRD showed no significant functional connection abnormality in the whole brain. There was no correlation between the *Z* scores of abnormal FC regions and any clinical data in patients.

**Table 3:** Brain areas showing altered FC with the right angular gyrus in patients with ESRD.

**Brain areas**	**Voxel**	**Peak T score**	**MNI coordinates**		
			**x**	**y**	**z**
Right angular gyrus	87	−6.2459	54	−60	24
Left precuneus	92	−5.0333	0	−60	21
Right superior frontal gyrus	182	−5.272	21	48	39
Right cuneus	56	4.9689	24	−78	9

**Table 4:** Brain area showing abnormal FC with the right precuneus in patients with ESRD.

**Brain areas**	**Voxel**	**Peak T score**	**MNI coordinates**		
			**x**	**y**	**z**
Left supramarginal gyrus	84	4.9041	−57	−21	33

## Discussion

The results of ALFF and FC analysis indicated that some important areas of DMN in hemodialysis patients with ESRD have altered spontaneous brain activity and functional integration impairment. The DMN brain areas are responsible for brain functions such as memory, attention, motion, and language and emotion management (32). The findings of ALFF were significantly correlated with neuropsychological and blood biochemical data. These findings indicated that taking precautions against neurodegeneration is necessary for hemodialysis patients with ESRD.

In our research, relative to the control group, the ALFF values of the right precuneus and RAG were decreased, but increased in the right precentral gyrus in the patient group. As a vital component of the DMN, the precuneus contributes to self-processing, the extract of episodic memory, and visuospatial imagination (33). The AG is one of the two parts of the IPL, and a reduction in its activity might indicate an impaired language and number processing, reasoning, and memory function (34). The precentral gyrus, also known as the primary motor cortex, is a very important structure involved in executing voluntary motor movements. Mu et al. found that patients with ESRD with Restless legs syndrome showed reduced gray matter volume of bilateral precentral gyrus when compared with the HCs (35). But in our study, the ALFF values of the right precentral gyrus were increased, which may suggest that the damage of the precentral gyrus was in the early stage and the compensative effect of spontaneous brain activity. Our findings of ALFF analysis are supported by the previous researches in patients with ESRD (15, 22–24, 36). Liang et al. revealed that patients with ESRD with minimal nephron-encephalopathy showed decreased ReHo in the right IPL, medial frontal cortex, and left precuneus, and altered ReHo values in some brain regions were correlated with neuropsychological tests and serological results (15). Chen et al. found that hemodialysis patients had aberrant ALFF in the DMN regions, such as the left parahippocampus/hippocampus, right precentral/postcentral gyrus, and precuneus (22). These results suggested that the spontaneous brain activity abnormalities in the DMN regions of patients of ESRD are well-repeatable. Therefore, we considered that the findings obtained by selecting abnormal ALFF regions as seeds for FC analysis are reliable.

In many neuropsychological tests, a lower score on MoCA was found in the patient group relative to HC. This may indicate that mild cognitive impairment had developed in patients in ESRD who were undergoing hemodialysis. Unexpectedly, after being corrected by multiple comparisons, MoCA scores exhibited no correlation with the ALFF values in the aforementioned brain regions. However, the correlation coefficient between the ALFF values of the right precuneus and the LTT results was negative in patients with ESRD. This might indicate that the altered spontaneous brain activity of the precuneus might affect the psychomotor speed and visuospatial ability of hemodialysis patients. Our results were similar to those of previous studies; Gu et al. also found that the MoCA scores were significantly lower in the ESRD group, and the ALFF values of the bilateral anterior cingulate gyri were positively correlated with the MoCA scores (37). Luo et al. found that the relationship between LTT scores and the ALFF values of the bilateral middle temporal gyri and cuneus were negative in peritoneal dialysis patients with ESRD (24). In combination with the aforementioned results, we inferred that abnormal spontaneous brain activity may be related to cognitive impairment in hemodialysis patients with ESRD.

Importantly, we found that the ALFF values of the right precentral gyrus were negatively correlated with Hb. Some previous studies have shown that anemia is associated with cognitive impairment in patients with ESRD (22, 24, 36). After treatment of anemia, the neuropsychological performance was improved (38). Our study further confirmed that correcting anemia in patients with ESRD may be an effective measure of neuroprotection. Because of the renal failure, the serum creatinine and urea levels were significantly increased in patients with ESRD. Previous researchers have found that the serum creatinine and urea levels were correlated with altered spontaneous brain activity and functional integration (15, 25, 39, 40). However, in our study, serum creatinine and urea levels showed no correlation with ALFF values or *Z* scores of abnormal FC in the brain regions. These inconsistent results may be attributed to the small sample size and different methodology.

Another important finding was that the ESRD groups showed decreased connectivity between the RAG with the left precuneus and RSFG, and the connectivity within the RAG was weak. The decreased connectivity may be because of the interruption of neuronal connection in the brain networks, and the dysfunction of functional integration in the selected areas of the DMN that might be related to cognitive impairment (41–43). These results indicated that the precuneus and AG have severer dysfunction in hemodialysis patients with ESRD, and might lead to several neurocognitive dysfunctions, including attention disorder, slowly processing speed, abnormal executive function, motor function, and memory (44). In contrast, enhanced connectivity between the RAG and right cuneus, and the right precuneus and left supramarginal gyrus was found in the ESRD group. The cuneus plays an important role in visual spatial processing, which is responsible for modifying and transmitting visual information to the extrastriate cortex (45). The meaning of increased connectivity strength might be a stronger synchronous BOLD fluctuation that might result from loss of the inhibitory influence (46). The second explanation of increased FC between the RAG and right cuneus might be an early compensatory or adaptive response to impaired vision. Patients on dialysis usually suffer from visual impairment (47, 48). However, the finding of our study was partly different from previous FC studies. Chen et al. found that enhanced FC between the precuneus, right inferior parietal lobule, and RAG in hemodialysis patients when compared with non-hemodialysis subjects (25). These results indicated that patients with ESRD have functional compensation. This might be related to different selected ROI methods. They defined reduced ReHo brain regions as seeds for FC analysis, but we chose the abnormal ALFF regions. Compared with the ReHo algorithm, ALFF can reflect the amplitude of spontaneous brain activity directly (17). Another reason may be the small sample size. Our results suggested that functional integration impairment and functional reorganization coexist in hemodialysis patients with ESRD.

We acknowledge that some limitations existed in our study. First, the sample size was small, which may have an adverse effect on the statistical analysis and reduced the persuasiveness of our research results. Second, we used a cross-sectional study; however, it is necessary to explore the changes in brain activity in patients with ESRD before and after renal transplantation by a longitudinal study. Third, we did not collect the results of blood biochemical tests for HCs, and the health status obtained by inquiry showed a lack of objective evidence. At last, the influence of blood pressure and the etiology of nephropathy on brain function in hemodialysis patients with ESRD was not assessed in our study.

## Conclusion

Using resting-state ALFF and FC analysis, our study showed that the hemodialysis patients with ESRD had serious abnormalities of spontaneous brain activity and functional integration in the DMN regions, particularly in the AG and precuneus, which may be associated with cognitive impairment. These findings offer additional evidences to realize the possible underlying neuropathological mechanisms in hemodialysis patients with ESRD.

## Data Availability Statement

The original contributions presented in the study are included in the article/supplementary material, further inquiries can be directed to the corresponding authors.

## Ethics Statement

The studies involving human participants were reviewed and approved by the Ethics Committee of Guangdong Second Provincial General Hospital. The patients/participants provided their written informed consent to participate in this study. Written informed consent was obtained from the individual(s) for the publication of any potentially identifiable images or data included in this article.

## Author Contributions

HS, GJ, and XQ designed and carried out the experiment. The clinical data were collected by SM. The results of neuropsychological tests and MRI data were obtained and sorted out by ML, YY, and KH. HS, SF, and KH processed and analyzed the complete data. The manuscript was written by HS and GJ. All authors contributed to the article and approved the submitted version.

## Funding

This study was supported by the National Natural Science Foundation of China (Grant Nos. U1903120, 81771807, 81701111, and 81901731), the Science and Technology Planning Project of Guangdong (202002030234), and the National Natural Science Foundation of Guangdong Province (2018A0303130129).

## Conflict of Interest

The authors declare that the research was conducted in the absence of any commercial or financial relationships that could be construed as a potential conflict of interest.

## Publisher's Note

All claims expressed in this article are solely those of the authors and do not necessarily represent those of their affiliated organizations, or those of the publisher, the editors and the reviewers. Any product that may be evaluated in this article, or claim that may be made by its manufacturer, is not guaranteed or endorsed by the publisher.
